# Association Between Social Networking Service Use at Age 76 and Six-Year Frailty Trajectories in Community-Dwelling Older Adults

**DOI:** 10.3390/medsci14060603

**Published:** 2026-09-24

**Authors:** Norio Imai, Daisuke Homma, Takuya Yoda, Yoji Horigome, Reiko Murakami, Toshihide Fujii, Hiroyuki Kawashima

**Affiliations:** 1 Division of Musculoskeletal Medicine, Niigata University Graduate School of Medicine, Dentistry and Health Sciences, 1-757, Asahimachi-dori, Chuo-Ward, Niigata 9518510, Japan; 2 Division of Orthopedic Surgery, Department of Regenerative and Transplant Medicine, Niigata University Graduate School of Medicine, Dentistry and Health Sciences, Niigata 9518510, Japan; 3 Department of Rehabilitation, Niigata Bandai Hospital, Niigata 9518850, Japan; 4 Department of Orthopedic Surgery, Agano City Hospital, Agano 9592092, Japan

**Keywords:** Kihon checklist, frailty, social networking service, longitudinal study, community-dwelling

## Abstract

Background: Frailty prevention is a critical public health priority in Japan’s rapidly aging society. Although the use of social networking services (SNSs) has increased, longitudinal evidence regarding its association with frailty remains limited. Methods: This population-based longitudinal observational study analyzed municipal survey data from Agano City, Japan. Community-dwelling residents aged 70 years at the baseline were followed for six years using the Kihon Checklist (KCL). Frailty status was classified as robust, pre-frail, or frail. SNS use and usage frequency were assessed at age 76. Results: Among 479 participants, 49.3% remained robust, while 10.4% showed improvements in frailty status over six years. SNS use at age 76 was associated with robust status and with having remained robust from age 70 to 76. Conclusions: These findings indicate that SNS use at age 76 was associated with concurrent frailty status and with having remained robust from age 70 to 76. However, the temporal direction of these associations cannot be determined. Further prospective studies are needed to clarify the temporal relationship between SNS use and frailty.

## 1. Introduction

Japan is experiencing rapid population aging, with the number of adults aged ≥75 years exceeding 20 million in 2023 [1]. Although life expectancy has increased substantially over recent decades, a persistent gap remains between life expectancy and healthy life expectancy [2]. In 2022, the gap between life expectancy and healthy life expectancy was 8.49 years for men and 11.63 years for women in Japan [2]. Consequently, extending healthy life expectancy has been positioned as a top national priority, with frailty prevention being recognized as one of its central strategies [3,4,5]. Frailty is a multidimensional syndrome that arises from the complex interplay of physical, psychological, and social factors; its progression greatly increases the risk of older individuals requiring long-term care [6,7]. Notably, frailty is potentially reversible; thus, its early identification and the initiation of appropriate interventions, including at the pre-frail stage, are essential to addressing this condition [6,7,8].

Declines in physical function and social activity have been reported to accelerate around the age of 75 years in Japan [9], highlighting the critical importance of early screening and preventive interventions for frailty during this transitional period [6,9,10,11]. In Agano City, Niigata Prefecture, community-based frailty assessments using the Kihon Checklist (KCL) have been conducted since 2011 to continuously monitor the health status of the city’s older residents. Using these longitudinal data, we previously reported that not only physical function but also social withdrawal, underweight status, reduced frequency of going outdoors, and depressive mood represent key factors associated with direct transitions from a robust state to frailty [12,13,14].

In recent years, the use of smartphones and social networking services (SNSs) has become increasingly common, even among older adults. Previous studies have reported associations between SNS use and several frailty-related domains in older adults, including social connectedness, physical activity, access to health-related information, and cognitive engagement [15,16,17,18,19,20,21,22]. These findings suggest that SNS use may be relevant to the multidimensional characteristics of frailty. However, most previous studies have been cross-sectional, and few have examined the association between SNS use and frailty trajectories derived from repeated assessments of the same community-dwelling older adults over an extended follow-up period. 

This study aimed to investigate longitudinal changes in frailty status among community-dwelling older adults in Agano City and to examine the association of SNS use at age 76 with concurrent frailty status and the preceding six-year frailty trajectory. We also aimed to discuss the implications of these findings for future community-based preventive strategies. We hypothesized that SNS use at age 76 would be associated with robust status at follow-up and with favorable frailty-related characteristics.

## 2. Methods

### 2.1. Study Setting and Participants

This longitudinal study was performed between 2019–2025 in Agano City, Niigata Prefecture, Japan. It has an estimated population of ~39,200 residents and an aging rate of 35.6%, which exceeds the 34.3% for the prefecture [23]. As part of an ongoing municipal public health program, the city’s Health Promotion Division periodically administers the Kihon Checklist (KCL) to older residents to assess overall frailty status and better guide community-based preventive strategies aimed at preserving functional independence. This study was designed as a longitudinal observational study using repeated municipal survey data from community-dwelling older adults in Agano City. The study size was determined by the number of eligible residents in the target age group who participated in the municipal survey, rather than by an a priori sample size calculation. For the primary multivariable logistic regression analysis, 330 participants were included, with 236 outcome events and 94 non-events. The model included three prespecified predictors, providing approximately 31 observations in the smaller outcome category per predictor. The study population comprised community-dwelling residents of Agano City who were aged 70 years in 2019 and 76 years in 2025. Residents who did not respond to the KCL or provided incomplete responses to it were excluded from the analysis.

This longitudinal study specifically targeted community-dwelling residents who were exactly 70 years old at baseline. In Japan, individuals aged ≥65 years are generally classified as older adults, with those aged ≥75 years representing an important age group in the healthcare system. Accordingly, the present cohort provided an opportunity to examine health changes across the transition from age 70 to beyond age 75.

No participants were excluded based on trauma history or major comorbid conditions. All of Agano City’s residents have been previously informed that anonymized municipal survey data may be used for academic research conducted by Niigata University.

### 2.2. Kihon Checklist

The KCL is a standardized self-administered questionnaire consisting of 25 binary (yes/no) items covering seven functional and psychosocial domains (Table 1): (1) activities of daily living, (2) physical function, (3) nutritional status, (4) oral function, (5) social engagement and confinement, (6) cognitive function, and (7) depressive symptoms [12,13,14,24,25]. Each affirmative response is scored as one point, resulting in a total score ranging between 0–25. The KCL has been widely validated as a screening instrument for frailty in Japanese community-dwelling older adults, and is routinely used in municipal health assessments [13,24,25].

Based on previously validated criteria, total KCL scores of 0–3 were classified as robust, 4–7 as pre-frail, and ≥8 as frail [24]. The same scoring criteria and classification thresholds were applied at both the 2019 and 2025 assessments. Participants with incomplete KCL responses were excluded; no missing-item imputation was performed. In the present study, the KCL was used as a validated frailty screening instrument reflecting multidimensional frailty-related vulnerability rather than as a diagnostic measure of frailty. Higher scores reflect greater functional vulnerability and a higher risk of requiring support or long-term care services. Frailty status was treated as an ordinal categorical variable (robust, pre-frail, or frail). This study evaluated changes in the overall distribution of these categories between 2019–2025, as well as individual-level transition patterns between them.

### 2.3. Assessment of Social Networking Service Use

In the 2025 survey, participants were asked, “Do you use social networking services (SNSs), such as LINE, Facebook, Instagram, or X?” Responses were dichotomized as use or non-use. No specific recall period was specified in the survey question. Among the 154 participants who reported SNS use, frequency was categorized as approximately once per month (*n* = 21, 13.6%), approximately once per week (*n* = 35, 22.7%), or ≥2 times per week (*n* = 98, 63.6%). “Use” referred to any self-reported use of these services; the survey did not distinguish between active interaction, posting, messaging, or passive viewing. General internet use, smartphone ownership, and other forms of ICT use were not included in the operational definition of SNS use. SNS use was assessed only at the 2025 follow-up and was not assessed at baseline.

### 2.4. Statistical Analysis

All statistical analyses were performed by an independent biostatistician (DH, PhD) who was not involved in the formulation of the study hypothesis or design. They were conducted using SPSS Statistics version 28 (IBM Corp., Armonk, NY, USA). Continuous data were summarized as medians and interquartile ranges, whereas categorical variables were assessed using Chi-squared testing. A multivariable logistic regression analysis was performed among participants who were robust at baseline to examine the association between SNS use at age 76 and having remained robust from age 70 to 76. Having remained robust (robust-to-robust vs. robust-to-pre-frail/frail) was treated as the dependent variable, and SNS use at age 76 was the primary independent variable. Sex and baseline KCL score were selected a priori as clinically relevant covariates representing demographic characteristics and baseline frailty-related vulnerability, respectively. All variables were entered simultaneously using the forced-entry method. Model fit was assessed using the Hosmer–Lemeshow goodness-of-fit test and Nagelkerke R^2^, multicollinearity was assessed using variance inflation factors (VIFs), and model discrimination was assessed using the area under the receiver operating characteristic curve (AUC). In a secondary analysis, SNS use frequency was treated as an ordinal variable coded as 0 for non-users, 1 for approximately once per month, 2 for approximately once per week, and 3 for ≥2 times per week to assess a linear trend in its association with having remained robust. This model was also adjusted for sex and baseline KCL score. Changes in the distribution of frailty status between 2019 and 2025 were assessed using the Stuart–Maxwell test of marginal homogeneity to account for the paired categorical nature of the repeated measurements. Individual frailty trajectories were then defined according to transitions in frailty status across the two survey waves. These included the maintenance of robustness, progression from robustness to pre-frailty, progression from robustness to frailty, and any improvements from frailty to either pre-frailty or robustness.

The relationships between these frailty trajectories and SNS use, including both usage status and usage frequency, were examined using Chi-squared analyses. Bonferroni adjustment was applied to control for any inflation of type I error in comparisons involving multiple frailty categories, resulting in a corrected significance threshold of *p* < 0.0167 (0.05/3). This adjustment was restricted to analyses comparing frailty categories, whereas all other statistical tests were interpreted using a two-sided significance level of *p* < 0.05.

Standardized self-administered questionnaires and identical assessment procedures were used at both time points to reduce potential information bias.

Any changes in responses to the 25 KCL items between the baseline and follow-up surveys were classified into four patterns: yes→yes, yes→no, no→yes, and no→no. The relationships between these item-level transitions and SNS use status and frequency were then analyzed. Because the KCL item-level analyses involved multiple comparisons, the Benjamini–Hochberg false discovery rate (FDR) procedure was applied to control for multiplicity. These item-level analyses were considered exploratory.

In our residual analyses, any adjusted residuals that exceeded an absolute value of 1.96 were considered indicative of statistically significant deviations from expected frequencies.

### 2.5. Ethics Statement

This study received ethical approval from the Institutional Review Board of Niigata University (approval number: 2025-0293). The research was performed in compliance with applicable ethical standards and regulatory requirements. As the analysis was based on pre-existing municipal administrative records and did not involve any interventional procedures, the institutional review board waived the requirement for informed participant consent.

## 3. Results

### 3.1. Longitudinal Changes in Frailty Prevalence

In 2019, 754 residents of Agano City (388 women, 366 men) were eligible for participation in this study. A total of 619 returned the mailed questionnaire, of which 586 responses were considered valid after incomplete entries were excluded.

The same cohort was re-surveyed using the KCL in 2025. Complete responses at both time points were obtained from 479 individuals, corresponding to a follow-up rate of 81.7% among the 586 valid baseline respondents and an overall retention rate of 63.5% relative to the 754 initially eligible residents (Figure 1). In the attrition analysis, participants who did not complete the six-year follow-up had higher baseline KCL scores than those who completed the follow-up (median [IQR], 4 [1–8] vs. 2 [1–4], *p* < 0.001). Baseline frailty status also differed significantly between non-completers and completers (*p* < 0.001); the prevalence of frailty was higher among non-completers (27.1% vs. 7.1%), whereas the prevalence of robustness was lower (45.8% vs. 68.9%). No significant difference in sex distribution was observed (*p* = 0.075). Detailed reasons for non-completion at follow-up, including death, relocation, or institutionalization, were not available in the municipal dataset.

At both 70 and 76 years of age, the largest proportion of participants was classified as robust—at 68.9% and 57.4%, respectively. However, the proportions of pre-frailty and frailty increased at 76 vs. 70 years of age, from 24.0% and 7.1%, respectively (70 years), to 29.7% and 12.9%, respectively (76 years). The distribution of frailty status changed significantly between ages 70 and 76 (Stuart–Maxwell test, χ^2^ = 28.385, df = 2, *p* < 0.001) (Figure 2).

Across the six-year follow-up period, 49.3% of the participants remained in a robust state between 70–76 years of age. A proportion of 10.4% showed any degree of improvement in their frailty status (Table 2).

### 3.2. Association Between Social Networking Service Use and Frailty Status

At 76 years of age, 32.2% of the participants reported using SNSs. Among them, 63.7% reported using SNSs ≥ 2 times per week (Figure 3).

Our cross-sectional analyses at age 76 revealed that the prevalence of frailty was significantly lower among the SNS users vs. the non-users (*p* < 0.0167; Figure 4).

Among the 330 participants who were robust at age 70, 236 (71.5%) remained robust at age 76, whereas 94 (28.5%) transitioned to pre-frailty or frailty. The proportion who remained robust was 81.4% (136/167) among SNS users and 61.3% (100/163) among non-users. In the multivariable logistic regression analysis adjusted for sex and baseline KCL score, SNS use at age 76 was associated with having remained robust from age 70 to 76 (OR 2.578, 95% CI 1.459–4.556, *p* = 0.001; Table 3). The model showed no evidence of poor fit (Hosmer–Lemeshow test, *p* = 0.208; Nagelkerke R^2^ = 0.160), no problematic multicollinearity was observed (all VIFs ≤ 1.010), and the AUC was 0.712 (95% CI, 0.655–0.769). In a secondary analysis, SNS use frequency at age 76 showed a significant graded cross-sectional association with having remained robust from age 70 to 76 after adjustment for sex and baseline KCL score (adjusted OR per one-category increase, 1.303; 95% CI, 1.053–1.611; *p* for trend = 0.015). In an exploratory analysis, there was no evidence that the association between SNS use at age 76 and having remained robust differed by sex (*p* for interaction = 0.528).

The analysis included 330 participants who were robust at age 70; 236 remained robust and 94 transitioned to pre-frailty or frailty by age 76. Having remained robust (robust-to-robust vs. robust-to-pre-frail/frail) was the dependent variable. SNS use at age 76 was the primary independent variable, with sex and baseline KCL score included a priori as covariates. All variables were entered simultaneously using the forced-entry method. Hosmer–Lemeshow test, *p* = 0.208; Nagelkerke R^2^ = 0.160; AUC = 0.712 (95% CI, 0.655–0.769). All VIFs were ≤1.010.

### 3.3. Changes in Individual Kihon Checklist Items and Their Association with Social Networking Service Use

In the exploratory item-level analyses, several KCL response transitions showed nominal associations with SNS use at an unadjusted *p* < 0.05 (Table 4). However, after correction for multiple testing using the Benjamini–Hochberg false discovery rate procedure, none of the item-level associations remained statistically significant.

## 4. Discussion

This study demonstrated that SNS use at age 76 was associated with robust status and with selected six-year frailty trajectories among community-dwelling older Japanese adults. In exploratory trajectory analyses, SNS use at age 76 was associated with having remained robust from age 70 to 76. SNS use frequency at age 76 also showed a graded cross-sectional association with having remained robust over the preceding six years.

These findings suggest that both SNS use and the degree of engagement with SNS platforms are associated with robustness among older adults. The graded cross-sectional association observed for SNS use frequency should be interpreted cautiously because SNS frequency was assessed only at age 76.

Several mechanisms have been proposed to explain previously reported associations between SNS use and frailty-related health, including social connectedness, cognitive stimulation through information-seeking behaviors, and self-management via digital health tools [3,19,21,26,27]. Large-scale cohort studies conducted in Japan have also reported associations between information and communication technology (ICT) use and higher levels of social participation, as well as better health-related outcomes, among older adults [28]. Unlike many previous cross-sectional studies, the present study relates SNS use assessed at age 76 to frailty trajectories derived from repeated KCL assessments over six years. However, because SNS use was assessed only at follow-up, the present findings do not establish a longitudinal exposure–outcome relationship. By following the same individuals over a six-year period, our analysis captured dynamic frailty trajectories that encompassed remaining robust, progression to pre-frailty or frailty, and improvements in frailty status. This approach facilitates a more nuanced understanding of frailty as a dynamic process rather than a static condition.

Furthermore, by evaluating SNS use not only as a binary variable, but also according to usage frequency, our findings suggest that the relationship between SNS engagement and frailty may depend on the frequency of use. Accordingly, our multivariable analyses were designed to evaluate associations after adjustment for sex and baseline KCL score rather than to establish causal pathways.

Exploratory item-level analyses identified several nominal associations between SNS use and KCL response transitions; however, none remained statistically significant after FDR correction. Therefore, these findings should not be interpreted as evidence of specific mechanisms linking SNS use with physical, social, cognitive, or nutritional domains. Moreover, “yes-to-yes” and “no-to-no” transitions represent persistence of the same response rather than improvement, and their clinical meaning depends on the direction of scoring for each KCL item. Therefore, the item-level findings should be interpreted cautiously, particularly given the large number of comparisons and the absence of statistically significant associations after FDR correction. Further prospective studies are needed to investigate these potential pathways.

Previous studies have suggested that excessive smartphone or screen use may be associated with sedentary behavior and physical inactivity [29,30], musculoskeletal symptoms and impaired balance or gait [31,32], and sleep disturbances [33,34]. Excessive smartphone use has also been reported to be associated with physical frailty [30]. However, the present study did not assess the duration of SNS use, and these potential adverse effects could not be evaluated.

The present findings support further investigation of digitally supported approaches but do not establish their effectiveness, feasibility, equity, or safety as interventions for frailty prevention. However, older adults often experience difficulties using ICT devices related to operational complexity, visual burden, or technology-related anxiety [35,36,37].

This study was subject to certain key limitations worth noting. First, the sample size was relatively modest, at ~500 participants. Second, the age range of the participants was restricted to 70 years at the baseline and 76 years at the follow-up, because the surveys were conducted as part of a municipal project. This limited age heterogeneity may restrict the generalizability of our findings to other age groups. Third, as the study relied on questionnaire-based data, frailty resulting from specific diseases or traumatic events (e.g., strokes or fractures) may not have been fully captured. Fourth, participants who did not complete the follow-up had higher baseline KCL scores and a higher prevalence of frailty than completers, suggesting potential attrition and survivor bias. Reasons for follow-up non-completion were unavailable, precluding further assessment of whether death, relocation, institutionalization, or other factors contributed to this attrition. In addition, because participation depended on questionnaire response, responder and healthy-participant biases cannot be excluded. Consequently, the analytic cohort may have disproportionately represented healthier older adults and should not be considered representative of all 70-year-old residents of Agano City, potentially limiting the generalizability of our findings. Because the analyses were restricted to participants with complete data at both assessments, complete-case selection may also have contributed to selection bias. Fifth, frailty trajectories between 70 and 76 years of age were evaluated in relation to SNS use, which was assessed only at age 76 and not at baseline. Therefore, SNS use cannot be assumed to have preceded the observed frailty trajectories, and the temporal direction of these associations cannot be determined. Reverse causation and a healthy-user effect are possible; individuals who remained healthier, more socially active, better educated, or more digitally literate may have been more likely to use SNSs at age 76. Thus, these characteristics may partly or wholly account for the observed association rather than reflecting a beneficial effect of SNS use itself. Furthermore, SNS use was self-reported using a single dichotomous question, which may have resulted in exposure misclassification or differential reporting. We could not distinguish participants who had continuously used SNSs throughout the follow-up period from those who initiated SNS use between the two surveys. In addition, SNS frequency was assessed using only three broad categories, and information regarding the duration of SNS use, specific platform used, purpose of use, and active versus passive engagement was unavailable. Therefore, the observed associations with SNS use and frequency should be interpreted cautiously. Sixth, SNS use may be influenced by ICT literacy and socioeconomic factors. Because educational attainment, socioeconomic status, and digital literacy were not comprehensively assessed, residual confounding related to the “digital divide,” defined as inequalities in access to and ability to use information and communication technologies, cannot be excluded [38]. Furthermore, digitally supported strategies may be less accessible to older adults with limited financial resources, sensory or cognitive impairments, low digital literacy, or limited internet access, and could therefore widen existing health disparities. Accordingly, if such approaches are evaluated in future studies, they should be considered as optional complements to, rather than replacements for, face-to-face health and social services.

Despite these limitations, this study has several notable strengths as well. A strength of this study is the repeated assessment of frailty using the KCL over six years in the same community-dwelling cohort. Moreover, the present study assessed SNS use frequency in addition to dichotomous use status and identified a graded cross-sectional association between SNS use frequency at age 76 and having remained robust from age 70 to 76.

The repeated assessment of the same community-dwelling residents over six years enabled characterization of individual frailty trajectories.

Further prospective research is warranted to clarify the temporal relationship between SNS use and frailty. Studies incorporating detailed assessments of SNS use—including its duration, type, purpose, and context—as well as interventional studies, are needed to determine whether SNS engagement has any role in subsequent frailty-related outcomes.

## 5. Conclusions

This study found that SNS use at age 76 was associated with concurrent frailty status and selected preceding six-year frailty trajectories among community-dwelling older adults. SNS use frequency at age 76 also showed a graded cross-sectional association with the preceding frailty trajectory. However, because SNS use was assessed only at follow-up, the temporal direction of these associations cannot be determined, and causal effects remain uncertain. Further prospective studies assessing SNS use before subsequent changes in frailty, as well as pragmatic intervention studies, are needed before SNS promotion can be considered as a frailty-prevention strategy.

## Figures and Tables

**Figure 1:**
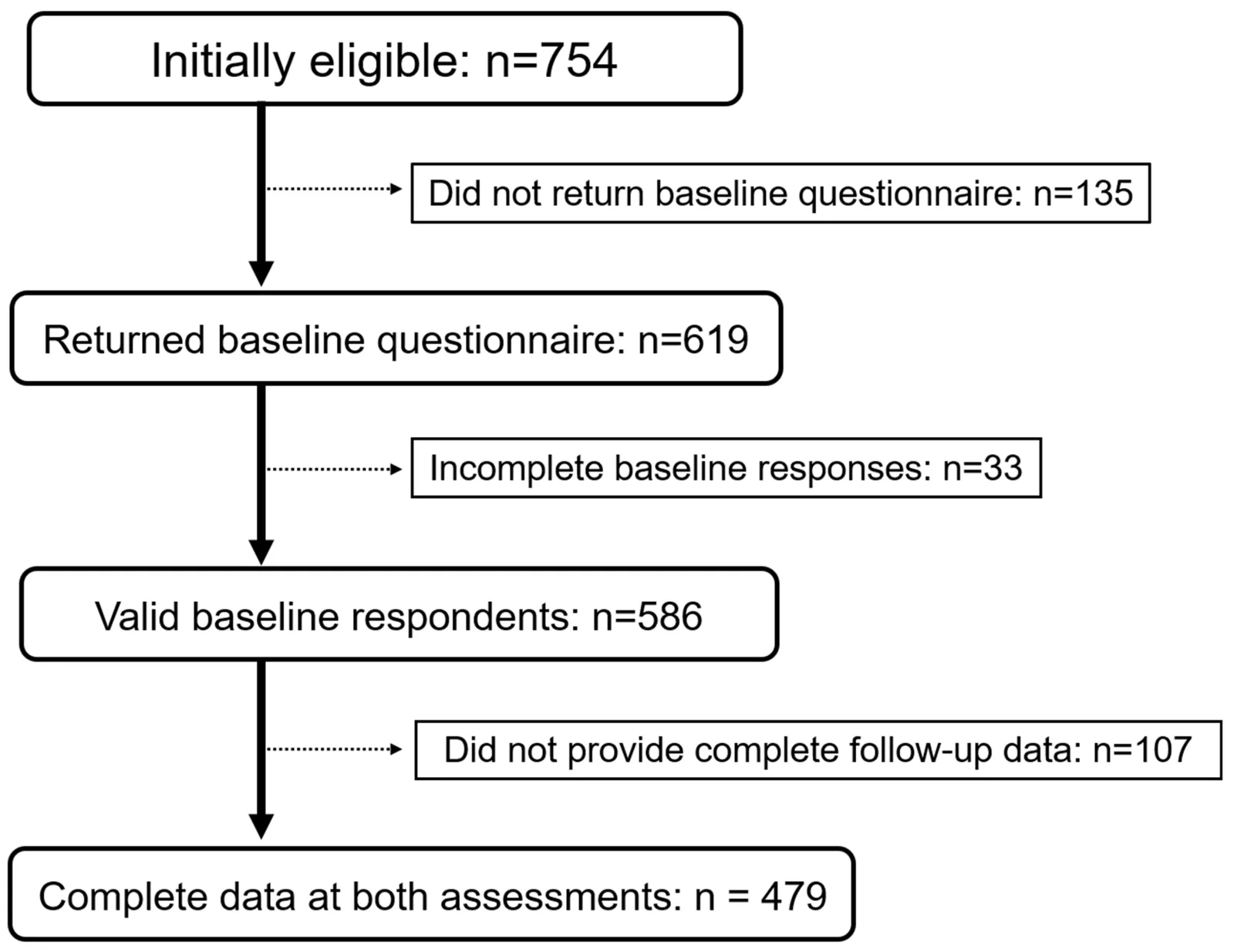
Flowchart of participant selection and follow-up. In 2019, 754 residents aged 70 years were eligible, of whom 619 returned the baseline questionnaire. After exclusion of 33 incomplete responses, 586 participants had valid baseline data. Of these, 479 provided complete data at both the baseline and six-year follow-up assessments, corresponding to a follow-up rate of 81.7% among valid baseline respondents and an overall retention rate of 63.5% relative to the initially eligible population. The remaining 107 baseline respondents did not provide complete follow-up data.

**Figure 2:**
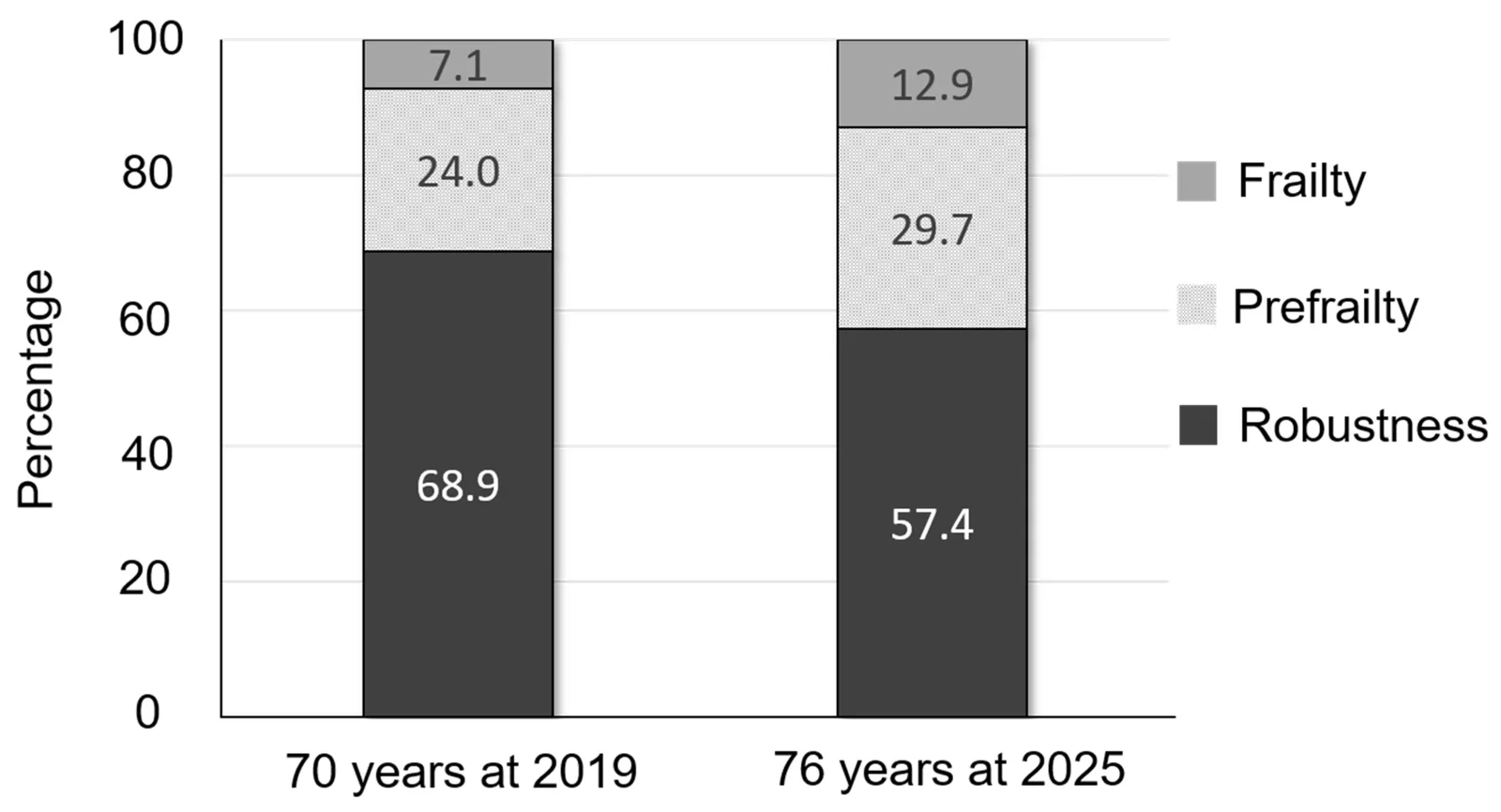
Distribution of frailty status among the study participants at ages 70 and 76 years. The proportions of participants classified as robust, pre-frail, and frail are shown for age 70 (2019) and age 76 (2025). The proportions of pre-frailty and frailty increased over the six-year period, and the overall distribution of frailty status differed significantly between the two assessments (Stuart–Maxwell test, *p* < 0.001).

**Figure 3:**
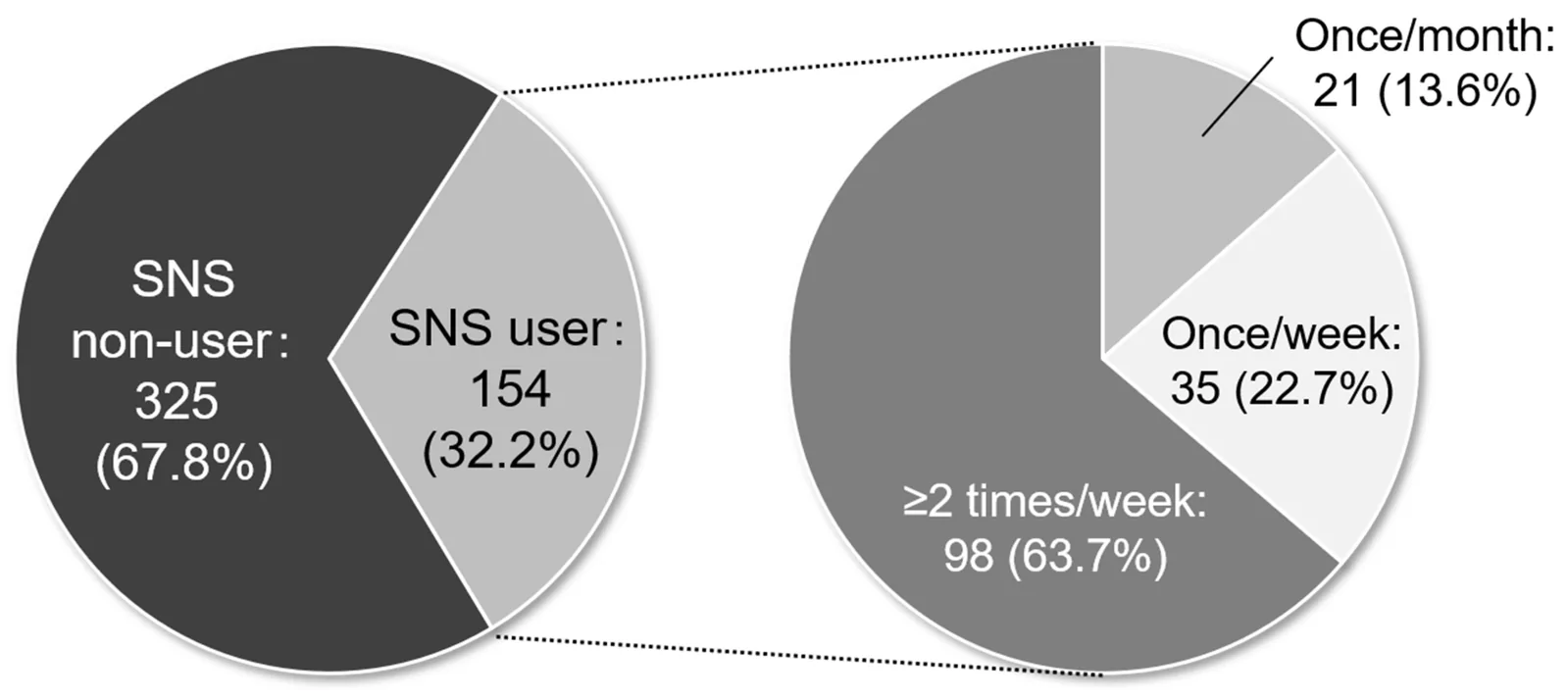
At 76 years of age, 154 (32.2%) participants reported using SNSs, whereas 325 (67.8%) were non-users. Among the 154 SNS users, usage frequency was categorized as approximately once per month (*n* = 21, 13.6%), approximately once per week (*n* = 35, 22.7%), and ≥2 times per week (*n* = 98, 63.7%).

**Figure 4:**
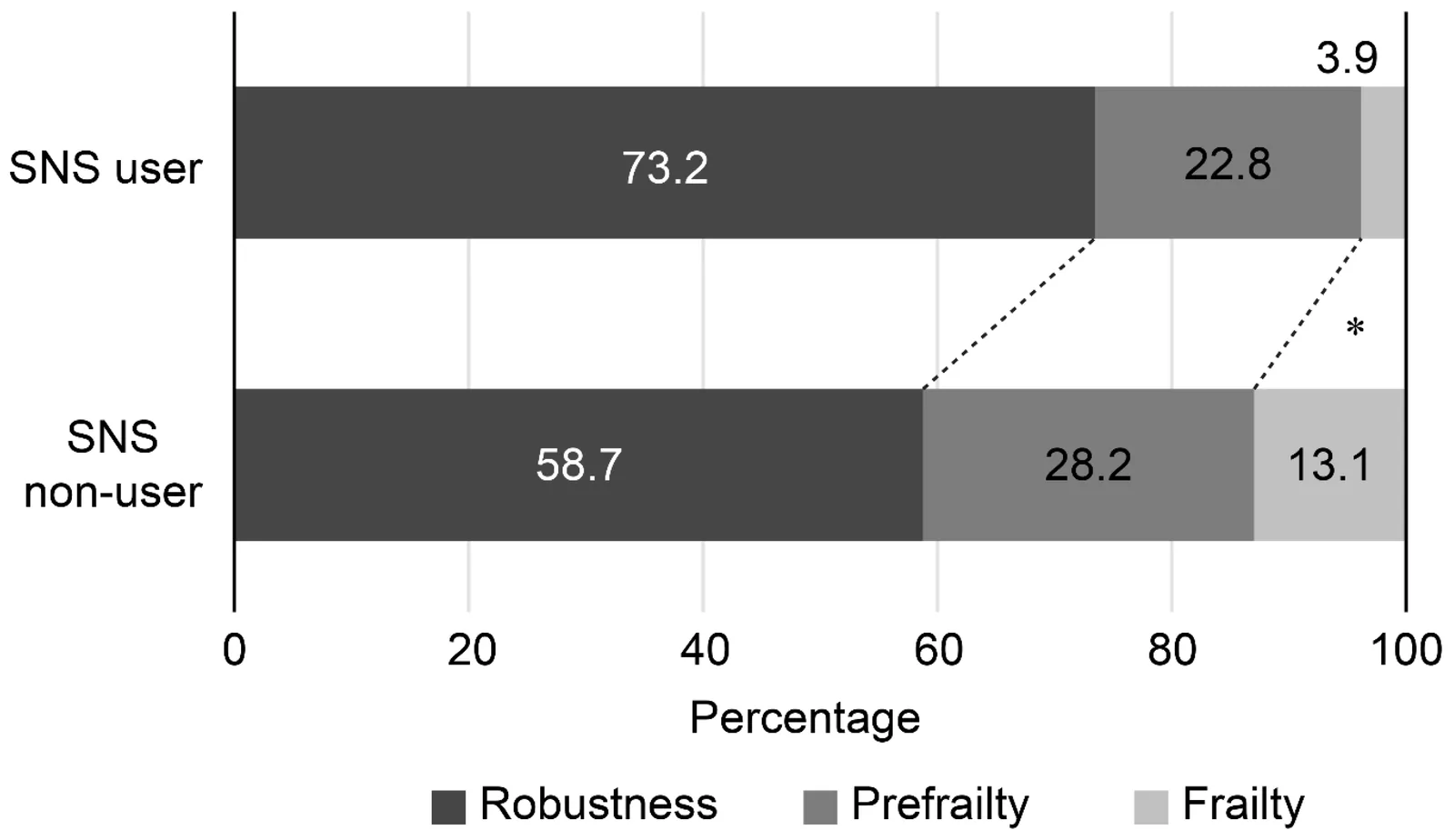
Association between SNS use and frailty status at age 76 years. The prevalence of frailty at age 76 was significantly lower among SNS users than among non-users (*p* < 0.0167, Bonferroni-corrected). * *p* < 0.05.

**Table 1:** Items of the Kihon Checklist [25].

No.	Item	Score	
1.	Do you go out by bus or train by yourself?	0. Yes	1. No
2.	Do you go shopping to buy daily necessities by yourself?	0. Yes	1. No
3.	Do you manage your own deposits and savings at the bank?	0. Yes	1. No
4.	Do you sometimes visit your friends?	0. Yes	1. No
5.	Do you turn to your family or friends for advice?	0. Yes	1. No
6.	Do you normally climb stairs without using handrails or walls for support?	0. Yes	1. No
7.	Do you normally stand up from a chair without any aids?	0. Yes	1. No
8.	Do you normally walk continuously for 15 min?	0. Yes	1. No
9.	Have you experienced a fall in the past year?	1. Yes	0. No
10.	Do you fear falling while walking?	1. Yes	0. No
11.	Have you lost 2 kg or more in the past 6 months?	1. Yes	0. No
12.	Height: cm, weight: kg, BMI: kg/m^2^ If BMI is less than 18.5 kg/m^2^, this item is scored	1. Yes	0. No
13.	Do you have any difficulties eating tough foods compared to 6 months ago?	1. Yes	0. No
14.	Have you choked on your tea or soup recently?	1. Yes	0. No
15.	Do you often experience having a dry mouth?	1. Yes	0. No
16.	Do you go out at least once a week?	0. Yes	1. No
17.	Do you go out less frequently compared to last year?	1. Yes	0. No
18.	Do your family or your friends point out your memory loss? E.g., “You always ask the same question over and over again”?	1. Yes	0. No
19.	Do you make a call by looking up phone numbers?	0. Yes	1. No
20.	Do you find yourself not knowing today’s date?	1. Yes	0. No
21.	In the last two weeks have you felt lack of fulfilment in your daily life?	1. Yes	0. No
22.	In the last two weeks have you felt a lack of joy when doing the things you used to enjoy?	1. Yes	0. No
23.	In the last two weeks have you experienced difficulty in doing what you could do easily before?	1. Yes	0. No
24.	In the last two weeks have you felt helpless?	1. Yes	0. No
25.	In the last two weeks have you felt tired without a reason?	1. Yes	0. No

**Table 2:** Transitions among frailty statuses in the study participants between 2019 (aged 70 years) and 2025 (aged 76 years).

		Aged 76 Years (2025)		
		Robustness	Pre-Frailty	Frailty
Aged 70 years (2019)	Robustness	236 (49.3%)	74 (15.4%)	20 (4.2%)
	Pre-frailty	34 (7.1%)	57 (11.9%)	24 (5.0%)
	Frailty	5 (1.0%)	11 (2.3%)	18 (3.6%)

**Table 3:** Multivariable logistic regression analysis of factors associated with having remained robust from age 70 to 76.

Variable	Unadjusted OR	95% CI	*p*-Value	Adjusted OR	95% CI	*p*-Value
SNS use at age 76	2692	1.554–4.663	<0.001	2.578	1.459–4.556	0.001
Baseline KCL score	0.548	0.433–0.694	<0.001	0.554	0.435–0.704	<0.001
Sex	1.156	0.715–1.868	0.554	1.221	0.732–2.035	0.444

**Table 4:** Exploratory associations between SNS use at age 76 and Kihon Checklist response transitions.

Item	Response Transition	Unadjusted OR	95% CI	Unadjusted *p*-Value	FDR-Adjusted *p*-Value
Q1	Yes to Yes	4.026	1.194–13.576	0.025	0.240
Q3	Yes to Yes	2.184	1.051–4.539	0.034	0.240
Q4	Yes to Yes	2.361	1.112–4.681	0.025	0.240
Q5	Yes to Yes	3.028	1.821–6.012	0.005	0.135
Q5	No to Yes	0.472	0.265–0.848	0.012	0.143
Q6	Yes to Yes	3.147	1.992–6.633	0.004	0.135
Q8	Yes to Yes	1.615	1.065–2.539	0.026	0.240
Q10	Yes to Yes	1.645	1.051–2.574	0.031	0.240
Q12	Yes to Yes	1.681	1.066–2.712	0.035	0.240
Q13	Yes to Yes	0.672	0.459–0.983	0.044	0.282
Q17	No to No	2.481	1.201–3.968	0.009	0.135
Q17	Yes to No	0.348	0.243–0.605	0.006	0.135
Q21	No to No	2.648	1.320–3.881	0.010	0.135
Q23	No to No	3.146	1.956–6.205	0.004	0.135
Q23	Yes to No	0.369	0.253–0.846	0.005	0.135
Q24	No to No	1.488	1.017–2.179	0.042	0.282
Q24	Yes to Yes	0.512	0.265–0.990	0.043	0.282

The KCL item-level analyses were considered exploratory. *p*-values were adjusted for 100 item-level comparisons using the Benjamini–Hochberg false discovery rate (FDR) procedure. None of the associations remained statistically significant after FDR correction. OR, odds ratio; CI, confidence interval; FDR, false discovery rate.

## Data Availability

The original contributions presented in this study are included in the article/Supplementary Material. Further inquiries can be directed to the corresponding authors.

## Supplementary Materials

The following supporting information can be downloaded at: https://www.mdpi.com/article/10.3390/medsci14060603/s1, Supplementary Figure S1. Conceptual diagram illustrating the temporal relationship between frailty assessment and SNS use. File S1: STROBE Statement—Checklist of Items for Cohort Studies.
