# Supplementary material for: Association Between Social Networking Service Use at Age 76 and Six-Year Frailty Trajectories in Community-Dwelling Older Adults
Source: Med Sci (Basel). 2026 Sep 24;14(6):603. doi: 10.3390/medsci14060603 (PMC13707300; doi:10.3390/medsci14060603)
Supplement: Supplementary file 1 [file medsci-14-00603-s001.zip › Figure S1.pdf]

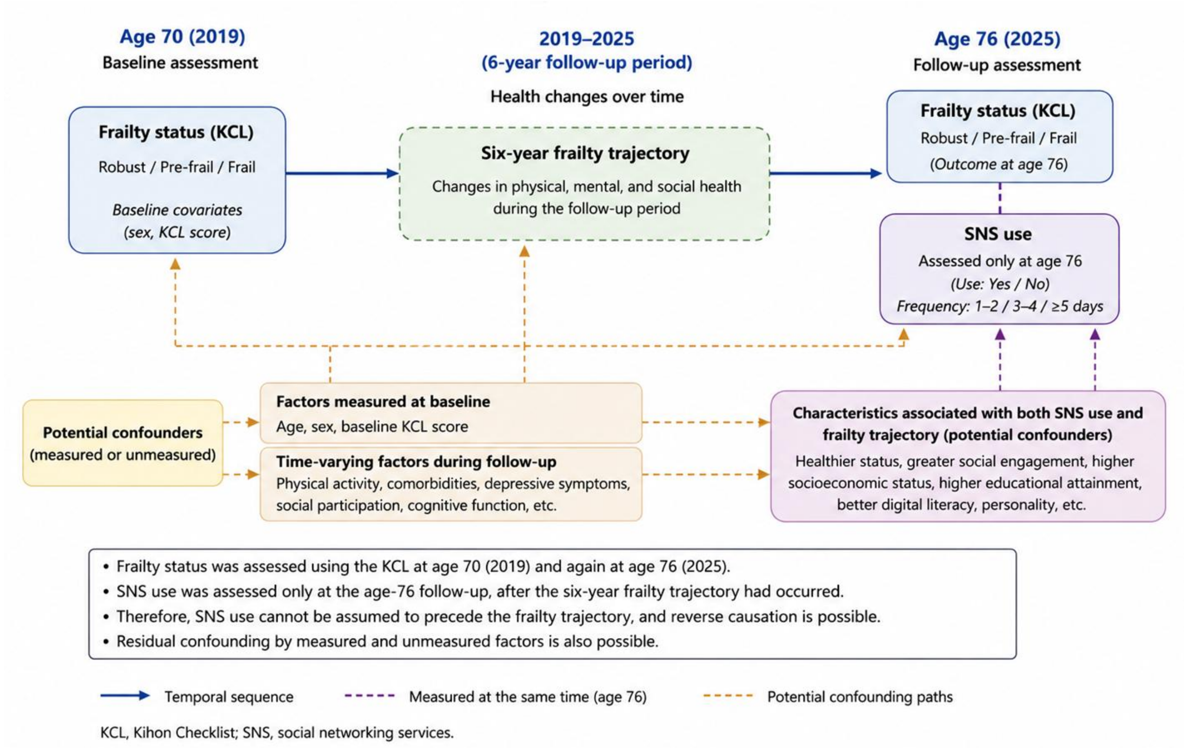

Supplementary Figure S1. Conceptual diagram illustrating the temporal relationship between frailty assessment and SNS use. Frailty status was assessed using the KCL at age 70 (2019) and age 76 (2025), whereas SNS use was assessed only at age 76. Therefore, SNS use cannot be assumed to precede the six-year frailty trajectory, and reverse causation and residual confounding are possible.
