# Supplementary material for: Association Between Social Networking Service Use at Age 76 and Six-Year Frailty Trajectories in Community-Dwelling Older Adults
Source: Med Sci (Basel). 2026 Sep 24;14(6):603. doi: 10.3390/medsci14060603 (PMC13707300; doi:10.3390/medsci14060603)
Supplement: Supplementary file 1 [file medsci-14-00603-s001.zip › File S1.pdf]

STROBE Statement—Checklist of Items for Cohort Studies

Completed for: Association Between Social Networking Service Use at Age 76 and Six-Year Frailty Trajectories in Community-Dwelling Older Adults

Section / topic	Item	STROBE recommendation	Where addressed in manuscript
Title and abstract	1(a)	Indicate the study's design with a commonly used term in the title or the abstract.	Title; Abstract (p. 1)
	1(b)	Provide in the abstract an informative and balanced summary of what was done and what was found.	Abstract (p. 1)
Introduction – Background/rationale	2	Explain the scientific background and rationale for the investigation being reported.	Introduction (pp. 1–2)
Introduction – Objectives	3	State specific objectives, including any prespecified hypotheses.	Introduction (p. 2)
Methods – Study design	4	Present key elements of study design early in the paper.	Methods 2.1 (pp. 2–3)
Methods – Setting	5	Describe the setting, locations, and relevant dates, including periods of recruitment, exposure, follow-up, and data collection.	Methods 2.1–2.3 (pp. 2–5)
Methods – Participants	6(a)	Give eligibility criteria and the sources and methods of selection of participants. Describe methods of follow-up.	Methods 2.1 (p. 3); Figure 1 (p. 7)
	6(b)	For matched studies, give matching criteria and number of exposed and unexposed.	Not applicable (not a matched cohort study).
Methods – Variables	7	Clearly define outcomes, exposures, predictors, potential confounders, and effect modifiers. Give diagnostic criteria, if applicable.	Methods 2.2–2.4 (pp. 3–6)
Methods – Data sources/measurement	8	For each variable of interest, give sources of data and details of methods of assessment; describe comparability of assessment methods if more than one group.	Methods 2.2–2.3 (pp. 3–5)
Methods – Bias	9	Describe any efforts to address potential sources of bias.	Methods 2.4 (pp. 5–6); Results 3.1 attrition analysis (p. 6); Discussion limitations (p. 12)
Methods – Study size	10	Explain how the study size was arrived at.	Methods 2.1 (p. 3): available municipal cohort; primary model n=330, 236 events and 94 non-events.
Methods – Quantitative variables	11	Explain how quantitative variables were handled in the analyses; describe groupings and why they were chosen.	Methods 2.2–2.4 (pp. 3–6): KCL categories; SNS frequency coded 0–3 for trend analysis.
Methods – Statistical methods	12(a)	Describe all statistical methods, including those used to control for confounding.	Methods 2.4 (pp. 5–6)
	12(b)	Describe any methods used to examine subgroups and interactions.	Exploratory sex-interaction analysis, if retained in final manuscript/response; otherwise not applicable.
	12(c)	Explain how missing data were addressed.	Methods 2.2: incomplete KCL responses excluded; no missing-item imputation. Complete-case follow-up described in Methods/Results.
	12(d)	If applicable, explain how loss to follow-up was addressed.	Results 3.1 (p. 6): attrition analysis; Discussion limitations (p. 12).
	12(e)	Describe any sensitivity analyses.	Methods/Results 3.2: secondary ordinal SNS-frequency trend analysis; other exploratory analyses as labeled.
Results – Participants	13(a)	Report numbers of individuals at each stage of the study.	Results 3.1 (p. 6); Figure 1 (p. 7)
	13(b)	Give reasons for non-participation at each stage.	Figure 1: baseline nonresponse and incomplete responses. Detailed reasons for follow-up non-completion were unavailable (Results 3.1).
	13(c)	Consider use of a flow diagram.	Figure 1 (p. 7)
Results – Descriptive data	14(a)	Give characteristics of study participants and information on exposures and potential confounders.	Results 3.1–3.2; Figures 2–3; Tables 1–2
	14(b)	Indicate number of participants with missing data for each variable of interest.	Methods 2.2 and Results 3.1; analyses used complete data as specified.
	14(c)	Summarize follow-up time.	Six-year follow-up, age 70 (2019) to age 76 (2025); Methods 2.1 and Results 3.1.
Results – Outcome data	15	Report numbers of outcome events or summary measures over time.	Results 3.1–3.2; Table 1; primary model: 236 remained robust, 94 transitioned.
Results – Main results	16(a)	Give unadjusted estimates and, if applicable, confounder-adjusted estimates and their precision; make clear which confounders were adjusted for and why.	Results 3.2; Table 2: unadjusted and adjusted ORs with 95% CIs; adjusted for sex and baseline KCL score.
	16(b)	Report category boundaries when continuous variables were categorized.	Methods 2.2–2.4: KCL 0–3, 4–7, ≥8; SNS frequency categories and ordinal coding.
	16(c)	If relevant, consider translating estimates of relative risk into absolute risk for a meaningful time period.	Results 3.2: among baseline-robust participants, 81.4% (136/167) of SNS users vs 61.3% (100/163) of non-users remained robust.
Results – Other analyses	17	Report other analyses done, e.g., subgroup, interaction, and sensitivity analyses.	Results 3.2–3.3: SNS-frequency trend; exploratory KCL item-level analyses with BH-FDR; sex interaction if retained.
Discussion – Key results	18	Summarize key results with reference to study objectives.	Discussion opening (p. 11)
Discussion – Limitations	19	Discuss limitations, including sources of potential bias or imprecision; discuss direction and magnitude of potential bias.	Discussion limitations (p. 12): attrition/survivor/responder/healthy-participant bias, temporal mismatch,

			reverse causation, exposure limitations, residual confounding/digital divide.
Discussion – Interpretation	20	Give a cautious overall interpretation considering objectives, limitations, multiplicity, similar studies, and other relevant evidence.	Discussion (pp. 11–13); item-level findings treated as exploratory and non-significant after FDR correction.
Discussion – Generalisability	21	Discuss the generalisability (external validity) of the study results.	Discussion limitations (p. 12): restricted age range, selected complete-case cohort, single municipality.
Other information – Funding	22	Give the source of funding and the role of the funders for the present study and, if applicable, for the original study.	Funding: None (p. 13).

Note: Page references are based on the revised manuscript dated 29 August 2026. The checklist should be updated if pagination changes in the final submitted version.
